# Synergistic Catalysis by “Polymeric Microzymes and Inorganic Nanozymes”: The 1+1>2 Effect for Intramolecular Cyclization of Peptides

**DOI:** 10.3389/fchem.2017.00060

**Published:** 2017-09-26

**Authors:** Zhiliang Chen, Börje Sellergren, Xiantao Shen

**Affiliations:** ^1^Key Laboratory of Environment and Health, Ministry of Education & Ministry of Environmental Protection and State Key Laboratory of Environmental Health (Incubation), School of Public Health, Tongji Medical College, Huazhong University of Science and Technology, Wuhan, China; ^2^Department of Biomedical Sciences, Faculty of Health and Society, Malmö University, Malmö, Sweden

**Keywords:** molecular imprinting, polymeric microzyme, peroxidase-like enzyme, inorganic nanozyme, synergistic catalysis, cyclization, disulfide peptides

## Abstract

In this work, we developed an efficient “molecularly imprinted polymer microzymes and inorganic magnetic nanozymes” synergistic catalysis strategy for the formation of disulfide bonds in peptides. The polymeric microzymes showed excellent selectivity toward the template peptide as well as the main reactant (linear peptide), and the Fe_3_O_4_ magnetic nanoparticle (MNP) nanozymes inhibited the intermolecular reaction during the formation of disulfide bonds in peptides. As a result, the integration of the two different artificial enzymes in one process facilitates the intramolecular cyclization in high product yields (59.3%) with excellent selectivity. Mechanism study indicates the synergistic effect was occurred by using a “reversed solid phase synthesis” strategy with an enhanced shift of reaction balance to product generation. We believe the synergistic catalysis by “polymeric microzymes and inorganic nanozymes” presented in the present work may open new opportunities in creation of multifunctional enzyme mimics for sensing, imaging, and drug delivery.

## Introduction

As a growth hormone inhibiting hormone, tetradecapeptide somatostatin (SST) was widely found in body organs of animals (e.g., the brain tissue, gastrointestinal, and pancreatic; Brazeau et al., 1974). Because of the presence of disulfide bond, SST is known as a more stable disulfide-rich cyclic peptide with a variety of physiological functions and medical values than linear peptides (Ginj et al., 2006). Generally, SST can inhibit the secretion of stomach and pancreas, stimulate mucus secretion, decrease portal venous pressure, relax biliary sphincter, relieve endotoxemia via stimulating the mononuclear macrophage system, inhibit the release of platelet activating factor, directly or indirectly regulate the cytokine chain to protect the cell (Hocart et al., 1998). Therefore, artificial synthesis of SST from chemical factory is of particular interest in pharmaceutical applications (Wu et al., 2001).

According to the literature, SST with disulfide bridges is usually synthesized via liquid-phase method or solid-phase method (Martín-Gago et al., 2014). In both methods, the final step is intramolecular cyclization of peptides between the two strategically selected cysteine residues (Cys). However, the general methods for this final step (the oxidation of Cys into disulfide bridges) suffered the following problem: the linear peptides were easily to form byproducts such as dimerization or oligomerization. To control the oxidation process and thus to obtain the desirable products, decreasing the concentration of linear peptide and adjusting the oxidization condition have been the main methods to currently improve the yield of the cyclization of peptides (Cheneval et al., 2014).

Decreasing the concentration of linear peptide is an efficient way to decrease the byproduct generation. However, this method also decreased the product amount. Recently, we presented an interfacial catalysis system using molecularly imprinted polymer (MIP) microgels (MGs) stabilized Pickering emulsions. This Pickering emulsion system enhanced the productivity while suppressed the formation of byproducts during the synthesis of SST. The MIP MGs, which possessed cavities in polymer matrix with affinity to a chosen “template” molecule, selectively promoted the intramolecular cyclization of SST (Shen et al., 2016). In the present work, we will further conduct intramolecular cyclization of the peptides in the solution by using the imprinted MGs as enzyme mimics (polymeric microzymes). Besides the suppressing of the byproduct formation, more advantages in cyclization of peptides using MIPs will be presented in this work.

Adjusting the oxidization condition is the second way to reduce the dimerization or oligomerization of the linear peptides during the formation of disulfide bonds. Traditionally, air, potassium ferricyanide, iodine, hydrogen peroxide (H_2_O_2_), dimethyl sulfoxide (DMSO), and thallium trifluoroacetate were often used as the oxidizing agents during the oxidation of Cys into disulfide bridges (Bulaj, 2005). However, these oxidizing agents seem to be very harsh compared to the natural oxidases, although the concentration of the linear peptides is very low. Therefore, an enzyme mimetic nanocatalyst (nanozymes), which can provide an oxidizing condition compared to the natural oxidases, will also be introduced into the formation of disulfide bonds in peptides in the present study.

Nanozymes, also named enzyme-like nanomaterials, can catalyze reactions under physiological conditions (Zhang et al., 2017). Because of the cost-effective and robust advantages, nanozymes display a broad spectrum of applications including biosensor, and chemical synthesis, environmental remediation, and disease treatment. Previous works have shown that various nanomaterials were discovered with oxidase, peroxidase, and superoxide dismutase mimicking activities (Zhang et al., 2017). Obviously, all these enzyme-mimicking nanomaterials have great potential in oxidating Cys into disulfide bridges. Among these nanozymes, Fe_3_O_4_ magnetic nanoparticle (MNP) is one of the best enzyme mimetic (peroxidase-like) catalysts. In 2007, the intrinsic enzyme mimetic activity of MNPs similar to that found in natural peroxidases was reported by Gao et al. (2007). Following this work, MNPs were widely utilized to oxidize organic pollutant in wastewater treatment. For example, using MNPs as a peroxidase mimetic, Wang et al. reported that Rhodamine B (RhB) was efficiently removed via a simple ultrasonic US-H_2_O_2_ system. It was found that Fe_3_O_4_ MNPs could catalyze the break of H_2_O_2_ to remove RhB in a wide pH range and their peroxidase-like activity was significantly enhanced by the ultrasound irradiation. Interestingly, the authors showed that an adsorption-desorption equilibrium of H_2_O_2_ was occurred on the MNP surface, and the catalytic efficiency was controlled by the adsorption of H_2_O_2_ (Wang et al., 2010).

Inspired by these works, herein we will use the peroxidase-like activity of the inorganic Fe_3_O_4_ MNPs to act as a new peroxidase-like material for the cyclization of linear peptide. In comparison with the traditional oxidizing reagents for disulfide bridge formation, the MNP nanozymes more like a natural oxidase, and the enzyme-like oxidizing reaction under near physiological condition facilitates the formation of SST. On the other hand, the oxidizing condition for L-SST is controllable since the oxidizing ability of the system depends on the adsorption of H_2_O_2_ on the MNPs.

Therefore, in this work we will propose a new method for low cost and effective cyclization of SST by integrating the MIP microzymes and MNP nanozymes. The polymeric microzymes and inorganic nanozymes will provide different advantages for the formation of disulfide bonds of linear peptides. During the cyclization, the linear peptides are activated simultaneously by two different artificial enzymes to conduct a single chemical transformation. This synergistic catalysis will further improve the reaction activity and catalytic selectivity.

## Materials and methods

### Materials

The monomers, *N*-isopropylacrylamide (NIPAm), *N*-tert-butylacrylamide (TBA), acrylic acid (AA), and *N*,*N'*-methylene bis(acrylamide) (MBA), were purchased from Sigma-Aldrich. *N*,*N*,*N'*,*N'*-Tetra-methyl-ethylenediamine (TEMED), ammonium persulfate (APS), and dithiothreitol (DTT) were supplied by Sigma-Aldrich. FeCl_3_•6H_2_O, FeSO_4_•H_2_O, NH_3_•H_2_O (25%), and oleic acid were obtained from Tianjing Chemical Reagent Company. SST (H-Ala-Gly-Cys-Lys-Asn-Phe-Phe-Trp-Lys-Thr-Phe-Thr-Ser-Cys-OH, molecular weight: 1638) and its linear structure (molecular weight: 1,640) and desmopressin (Mpr-Tyr-Phe-Gln-Asn-Cys-Pro-D-Arg-Gly-NH_2_, molecular weight: 1,069) and its linear structure (molecular weight: 1,071) was obtained from WuHan Moon Biosciences Co., Ltd. Reference somatostatin (rSST, Ser-Asn-Pro-Ala-Met-Ala-Pro-Arg-Glu-Arg-Lys-Ala-Gly-Cys-Lys-Asn-Phe-Phe-Trp-Lys-Thr-Phe-Thr-Ser-Cys, molecular weight: 2,375) was also obtained from WuHan Moon Biosciences Co., Ltd. Other chemicals were of reagent grade or higher.

### Synthesis of Fe_3_O_4_ MNPs (inorganic nanozymes)

The inorganic nanozymes were prepared following a same method in our previous work (Tang et al., 2012). In brief, 4.86 g of FeCl_3_•6H_2_O and 3.34 g of FeSO_4_•7H_2_O, and 40 mL of distilled water were homogenize and heated to 90°C. After the addition of ammonium hydroxide (12 mL) and oleic acid (0.8 mL), the reaction system was placed at 90°C for 3 h under magnetic stirring. The obtained oleic acid coated MNPs were washed with ethanol and distilled water, respectively. When the washing solution was neutral separation, the MNPs were dried under vacuum for 24 h. The MNPs were stored in a glass bottle (which was covered with an aluminum paper to avoid light illumination).

### Synthesis of MIP microgels (polymeric microzymes)

The polymeric microzymes were synthesized via the same method reported in our previous paper (Shen et al., 2016). Briefly, A homogenous solution was first obtained by mixing 20.7 μL of AA, 217.3 mg of NIPAm, 61.0 mg of TBA and 46.3 mg of MBA, 6.8 mg of SST template and 20 mL of PBS buffer (pH 7.4, 20 mM) together. The particles in the reaction system were removed through a 0.45 μm filter. After addition of 20 μL of APS solution (10%) and removal of the O_2_ in the system by nitrogen bubbling, the reaction system was placed at 50°C for 3 h under shaking. In the second step, 120 μL of APS solution (10%) and 60 μL of TEMED were added into the reaction solution. Following completion of initiator supplement, the polymerization system was again placed at 50°C for 1 h under shaking. The polymeric MGs were purified by dialysis using 1 L of pure water for 3 days, 1 L of water containing 3 mL of 4 M HCl for 3 days, and 1 L of pure water for 2 days, successively. The washing solution was changed more than four times per day.

The leakage of target peptide from MIP MGs was measured at room temperature by a spectrofluorometer (F-97 Pro, Shanghai Lengguang Technology Co. Ltd., China). The excitation and emission wavelength for SST were 280 and 356 nm, respectively. The washing step was finished when no SST was measured in the supernatant. The MIP MG solution was diluted with water to 9.0 mg mL^−1^ (dry polymer) for further application. The NIP MG solution was also generated in the absence of templates during the synthesis.

### Characterization

Magnetic property of the MNP nanozymes was tested with a vibrating sample magnetometer (ADE 4HF VSM). The morphology of the polymeric MGs was measured by a scanning electron microscope (Inspect SEM F50, FEI Company). The size distribution of the MNPs and the wet MGs was evaluated using dynamic light scattering (DLS) with a Coulter LS230 instrument (Beckman-Coulter Co. Ltd.). The particle concentration for both MNPs and MGs was 0.1 mg mL^−1^ during the testing.

### Binding and selectivity test

The molecular recognition ability of the MIP MGs was investigated also by incubating the polymeric MG solution (containing 5.4 mg of dry MGs) and SST (with different concentrations) in a 1.5 mL Eppendorf tube. After a 16 h-incubation at room temperature, the polymeric MGs were isolated by centrifugation for 15 min at a speed of 14,000 rpm. The SST concentration in the supernatant was then analyzed on a spectrofluorometer. The excitation and emission wavelength were 280 and 356 nm, respectively. The amount of SST bound to the polymeric MGS was calculated from the decreasing of the fluorescence intensity compared to the solution before binding. The equilibrium adsorption capacity (*q*_*e*_, mg g^−1^) of SST by the polymeric MGs is calculated via the following equation:

(1) $$\begin{array}{r} q_{e}=\frac{\left(C_{0}-C_{e}\right)\cdot v}{m} \end{array}$$

where *C*_0_ and *C*_e_ are the equilibrium concentration of SST (mg mL^−1^) before (initial) and after the adsorption, respectively. *v* and m are the volume of SST solution and the mass amount of the dry MGs, respectively.

To test the selectivity of MIP MGs, the binding of reference peptides (including L-SST, rSST, DDAVP, and MSH) was investigated. The concentrations of L-SST, rSST, and MSH were measured using a same method for SST. The concentration of DDAVP was determined using HPLC with a diode-array detector (Chen et al., 2016). The HPLC method for DDAVP followed a previous work (Christophersen et al., 2014).

### Catalysis study

5 mg of MNP nanozymes and 100 μL of 100 mmol L^−1^ H_2_O_2_ were mixed and placed at room temperature for 3 min. Another mixture (900 μL) containing L-SST (360 mmol L^−1^) and polymeric microzymes (5.4 mg mL^−1^) in PBS buffer (pH 7.4, 20 mM) was placed at room temperature for 30 min. The reaction started when these two systems were mixed together. As controls, cyclization of L-SST using MNP nanozymes, MIP microzymes, pure H_2_O_2_, H_2_O_2_ + MNP nanozymes, H_2_O_2_+ MIP microzymes, and H_2_O_2_ + MNP nanozymes + NIP MGs were carried out. After a certain time of reaction, the reaction solution were sampled and centrifuged to remove the MGs. The MGs were washed with three times using 0.5 mL of HCl solution (0.1 M). The first supernatant and the washing solutions were mixed together. The concentration of the product SST was analyzed using the HPLC according to a previous method (Shen et al., 2016). The concentration of thiol groups in L-SST was measured by Ellman's reagent. Typically, 0.5 mL of the mixture and 50 μL of Ellman's reagent stock solution were mixed and place at room temperature for 3 min. After the reaction, the absorbance of the derivative products was measured at 410 nm with a Shimadzu UV-2550 scan UV/vis spectrophotometer. The conversion rate of L-SST is the ratio between the consumption of the thiol groups and the initial amount of the thiol groups. MALDI MS analysis of the byproducts was carried out with a same method in our previous work (Shen et al., 2016).

## Results and discussion

### Characterization of materials

**Figure 1:**
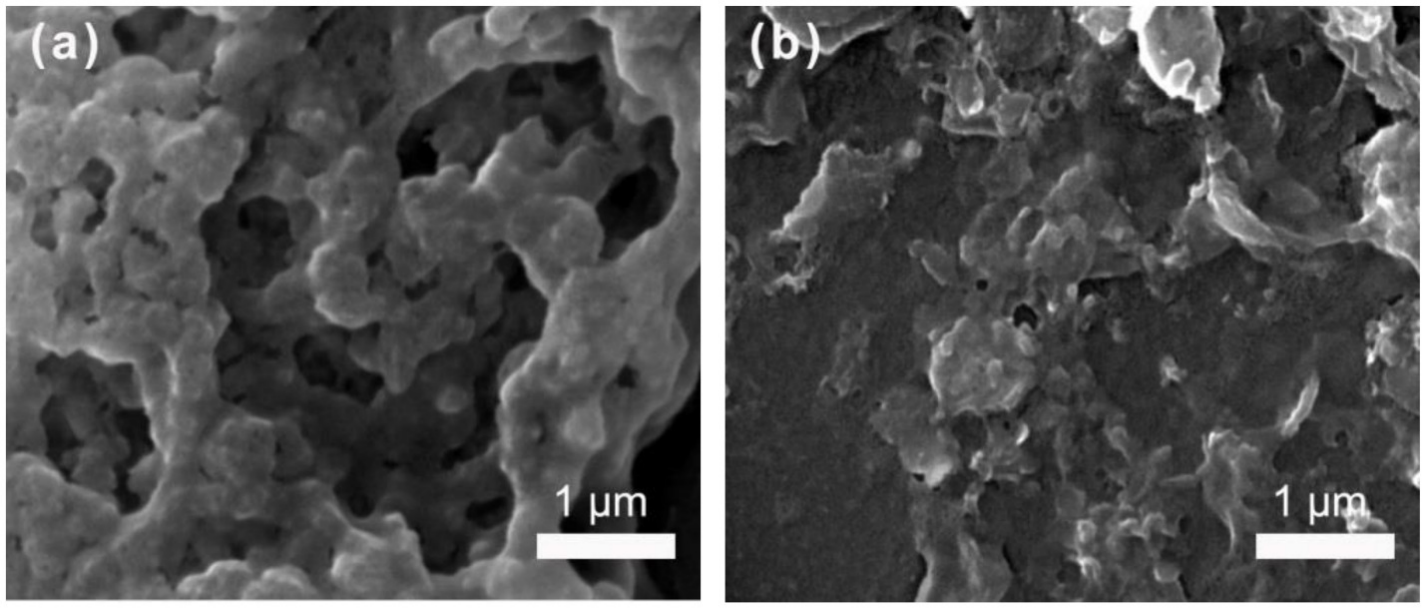
SEM images of MIP MGs **(a)** and NIP MGs **(b)** casted on a glass slide.

Precipitation polymerization with a programmed initiator change strategy was an efficient way for synthesis of MIP MGs (Meng et al., 2009). The morphology of the dry MGs was observed using a scanning electron microscope (SEM). It is seen in Figure 1 that the dry MIP and NIP MGs were both gel-like polymers. Utilizing SEM and DLS measurements, our previous work has shown that the MIP MGs owned a dry diameter of ~100 nm and a wet diameter of ~280 nm, respectively. Supposing the particles were spherical in shape, the swelling ratio of the MIP MGs were ~20. This high swelling character provides the MIP MGs with enough channels for peptide diffusion.

**Figure 2:**
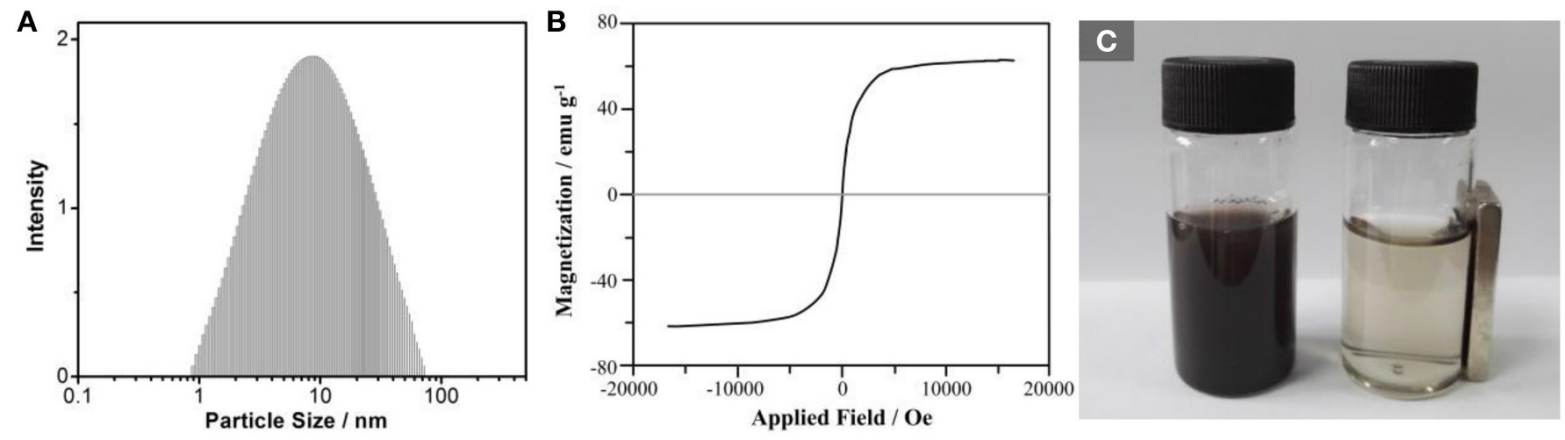
**(A)** DLS analysis of MNP nanozymes in methanol; **(B)** VSM measurement of MNP nanozymes; **(C)** Photographs of MNP nanozymes suspended in water in the absence (left) and in the presence (right) of an external magnetic field.

Our previous work has shown that the MNP nanozymes ranged from 10 to 20 nm by using a TEM analysis (Tang et al., 2012). Here, this size distribution was confirmed by using the DLS measurement in Figure 2A (~12 nm). Magnetic features of MNP nanozymes were recorded by VSM measurement. It is seen in Figure 2B that the MNP nanozymes revealed superparamagnetic activities, the saturation magnetization (Ms) values for MNP nanozymes is ~60 emu g^−1^. Figure 2C demonstrates that the MNP nanozymes could be facilely isolated by an external magnetic field.

### Binding profiles of polymeric microzymes

**Figure 3:**
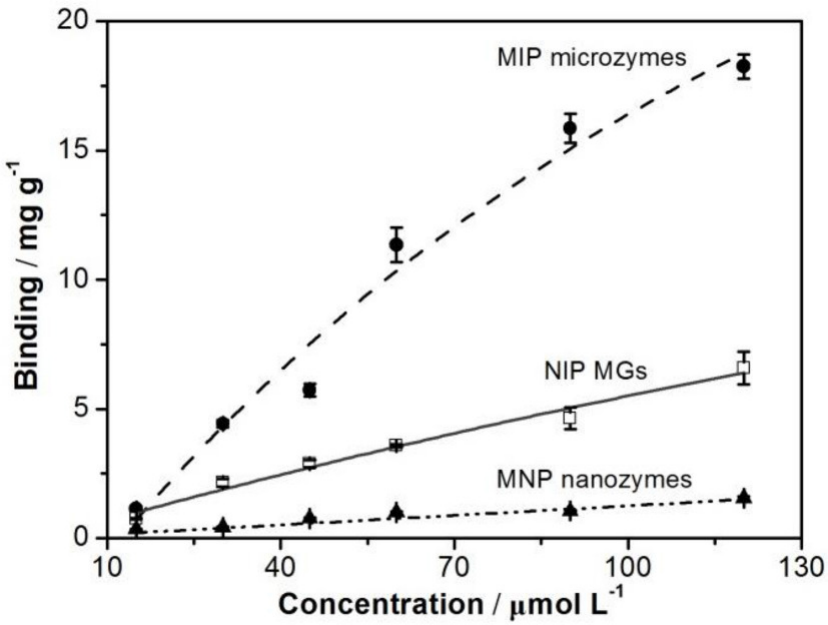
Binding isotherms of SST on MIP microzymes, NIP MGs, and MNP nanozymes. The particle concentration was 5.4 mg mL^−1^.

The SST recognition by the polymeric microzymes was studied by fluorescence spectrometry. Figure 3 shows the binding isotherm of SST (from 15 to 120 μmol L^−1^) on the MIP MGs. As controls, the binding isotherm of SST on the NIP MGs and the MNP nanozymes was also carried out. It is seen that the binding of SST by the MNP nanozymes was neglected. For both polymeric MGs, the binding capacity of SST enhanced with the increasing of the SST concentration. However, in comparison with the NIP MGs, MIP MGs displayed a much more template uptake.

In our previous work, four peptides including L-SST, reference somatostatin (rSST), desmopressin (DDAVP), and melanocyte stimulating hormone (MSH) have been selected as references to probe the selectivity of the polymeric microzymes. It is noted that L-SST and rSST are the analogs of the template SST, while DDAVP and MSH are not. Therefore, the selection of the four control peptides with different structure similarity was proper to investigate the recognition selectivity of the MIP MGs. The experimental data showed that the polymeric microzymes showed higher binding capacities toward SST, L-SST, and rSST than the NIP MGs. The tendency of the selectivity of the polymeric microzymes was in the order SST > L-SST > rSST > DDAVP > MSH, which might be because of structural similarity of these peptides (Shen et al., 2016). It is noted that the polymeric microzymes also showed selectivity to L-SST (the main reactant of the product), which will play a significant role during the cyclization of L-SST.

### Synergistic catalysis study

**Figure 4:**
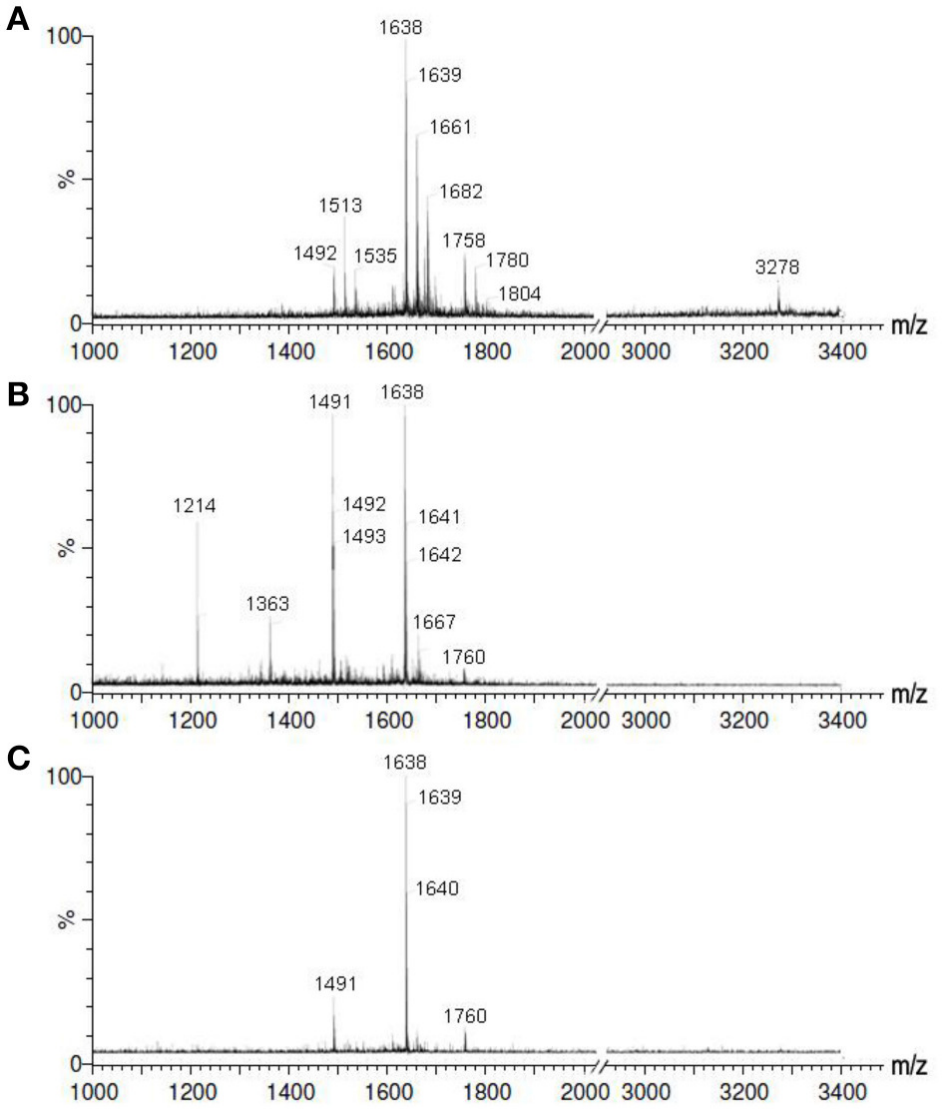
MALDI analysis of the SST and the byproducts after a 30-min reaction for H_2_O_2_, **(A)** H_2_O_2_ + MNP nanozymes, **(B)** and H_2_O_2_ + MNP nanozymes + MIP microzymes. **(C)** The peptide concentration in the water phase was 120 μM. m/z 1639: SST + H^+^; m/z 1491: SST lost Cys residue; m/z 1660 and 1661: SST + Na^+^; m/z 3278: SST dimer + H^+^.

The synergistic catalysis by the “polymeric microzymes and inorganic nanozymes” was conducted with respect to the disulfide formation of linear peptides. Firstly, the byproduct formation during the cyclization of linear peptides was investigated by MALDI analysis. In our previous work, we have demonstrated that the mixture systems by adding the oxidizing reagent to L-SST solution in the presence/absence of polymeric MGs showed high peptide dimer yields (Shen et al., 2016). This was also confirmed in Figure 4A when H_2_O_2_ was used as the oxidizing reagent instead of iodine (the data for pure H_2_O_2_, which was same to H_2_O_2_ + MIP microzymes, was not shown here). However, when MNP nanozymes were introduced into the oxidizing system, the peptide dimers were not observed in the systems of H_2_O_2_ + MNP nanozymes (Figure 4B) and H_2_O_2_ + MNP nanozymes + MIP microzymes (Figure 4C). It is noted that peptide dimers were not found also in the system of H_2_O_2_ + MNP nanozymes + NIP MGs (data was not shown here). Therefore, we conclude that the application of MNP nanozymes is an efficient way to inhibit the intermolecular reaction during the formation of disulfide bonds in peptides.

**Figure 5:**
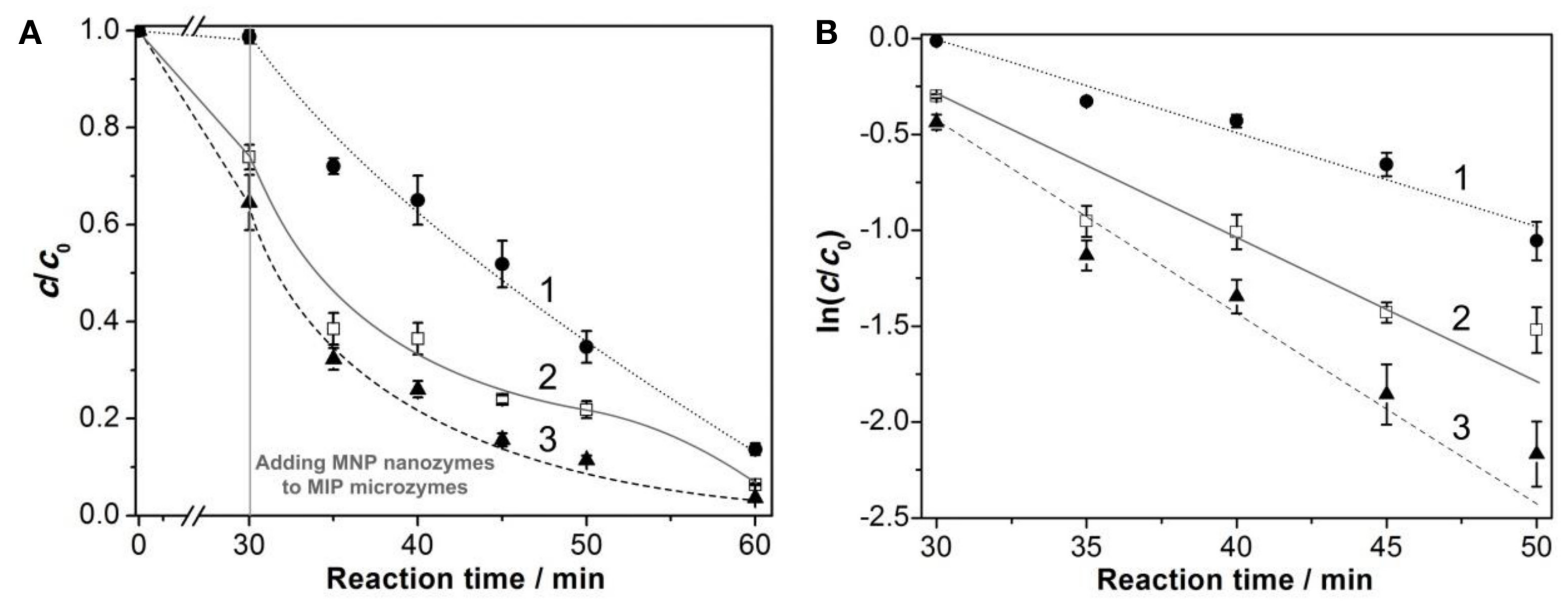
Time course of L-SST concentration **(A)** and cyclization kinetics for L-SST **(B)** in the presence of MNP nanozymes with or without polymeric microzymes. The MNP nanozymes were added to polymeric microzymes after an equilibrium time of 30 min. The pseudo-first-order constants for H_2_O_2_ + MNP nanozymes (1), H_2_O_2_ + MNP nanozymes + NIP MGs (2), and the H_2_O_2_ + MNP nanozymes + MIP microzymes (3) were 0.048, 0.058, and 0.084 min^−1^, respectively.

Secondly, the conversion of L-SST to cyclic peptide was investigated by measurement of thiol groups in L-SST using Ellman's reagent. It is found that the cyclization is inefficient in the absence of H_2_O_2_ for both MNP nanozymes and MIP microzymes (data were not shown here). For the other reaction systems, when H_2_O_2_ was used as the oxidizing reagent, the initial conversion efficiency increased with a finally complete conversion (Figure 5A). However, although the reaction systems of pure H_2_O_2_ and H_2_O_2_ + MIP microzymes showed higher initial conversion efficiency, but the systems yielded more by products (see Figure 4). Therefore, the reaction systems of pure H_2_O_2_ and H_2_O_2_ + MIP microzymes were not selected in the following text. According to the literature, the disulfide formation of linear peptides followed a pseudo-first-order kinetics (Shen et al., 2016). Using the pseudo-first-order model, the initial phase (0–30 min) of the data (for the systems with MNP nanozymes) in Figure 5A was plotted. The apparent rate constant *k* of the pseudo-first-order reaction was thus calculated and shown in Figure 5B. It is seen that the conversion tendency of the reaction systems were in the order: H_2_O_2_ + MNP nanozymes + MIP microzymes > H_2_O_2_ + MNP nanozymes + NIP MGs > H_2_O_2_ + MNP nanozymes. This result indicates the presence of MIP microzymes enhanced the conversion in the initial phase of the cyclization.

Thirdly, the yield of SST product from L-SST was investigated. As a control, the yield of DDAVP product from linear DDAVP was also studied (Table 1). For L-SST, the product yield for the system of H_2_O_2_ + MNP nanozymes + MIP microzymes was 59.3%, which was much higher than the system of H_2_O_2_ + MNP nanozymes + NIP microzymes (42.2%) and the system of H_2_O_2_ + MNP nanozymes (35.6%). However, when Linear DDAVP was use as linear peptide reactant, the system of H_2_O_2_ + MNP nanozymes + MIP microzymes showed a same product yield (no selectivity) to the H_2_O_2_ + MNP nanozymes + NIP microzymes. These experimental results demonstrate that the imprinted cavities enhanced selectively the cyclization of L-SST.

**Table 1:** Studies of the synergistic catalysis^a^.

**Entry**	**Peptide**	**Microzymes**	**Conversion %**	**Yield %**
1	L-DDAVP	No	>95	46.5
2		NIP	>95	48.4
3		MIP	>95	43.7
4	L-SST	No	>95	35.6
5		NIP	>95	42.2
6		MIP	>95	59.3

a *All systems contain H_2_O_2_ + MNP nanozymes*.

### Mechanism study

The above text has shown that, during the formation of disulfide bonds in peptides, the MNP nanozymes played an important role in inhibition of the intermolecular reaction, whereas the MIP microzymes selectively enhanced the product yields of SST. By combining the MIP microzymes and the MNP nanozymes together, synergistic catalysis occurred with an enhanced intramolecular cyclization of peptides and a decreased formation of intermolecular products. This synergistic catalysis by “polymeric microzymes and inorganic nanozymes” showed a clear 1+1>2 effect for the intramolecular cyclization of peptides.

**Figure 6:**
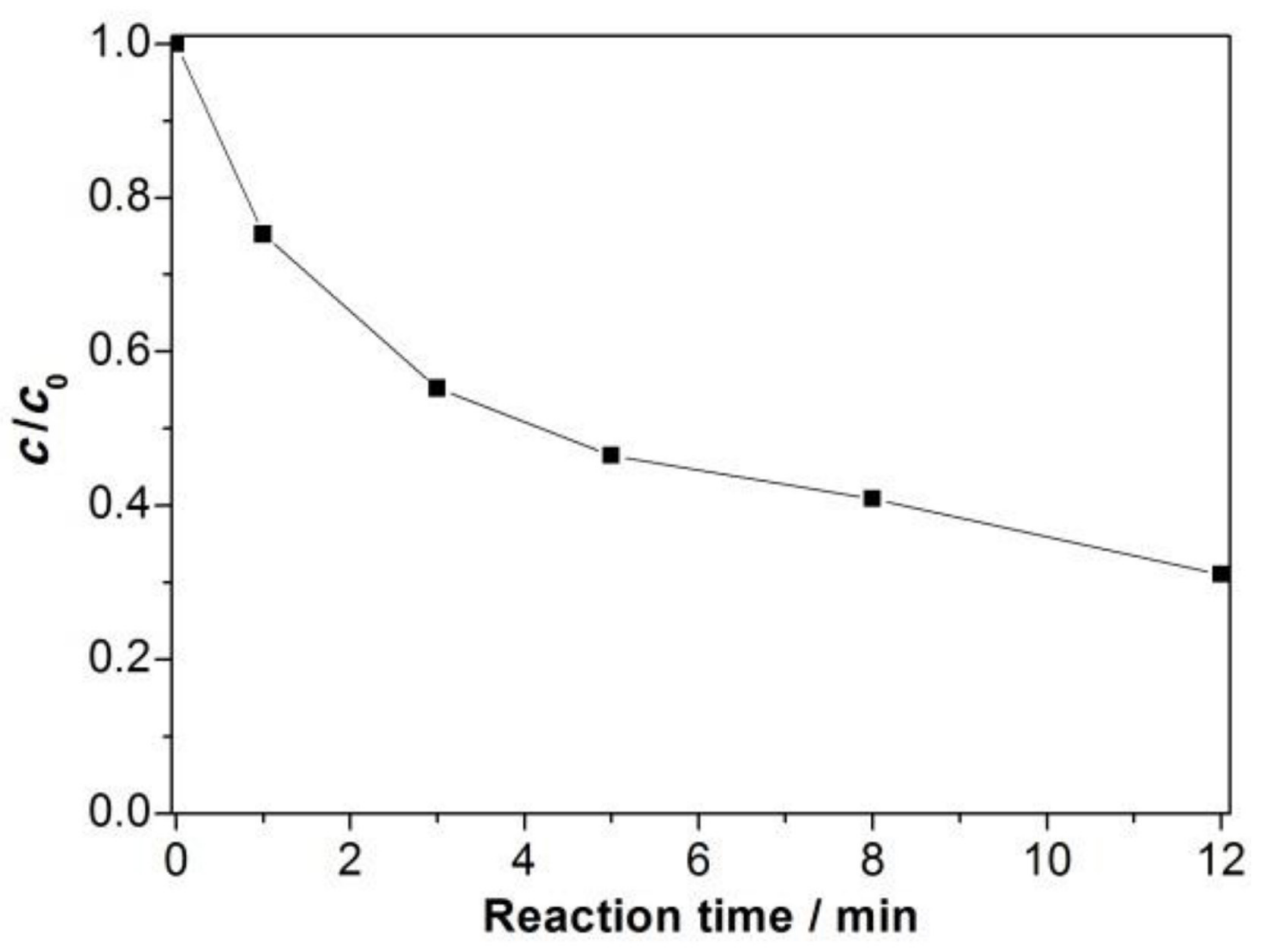
Time course of H_2_O_2_ concentration in the presence of MNP nanozymes (5 mg L^−1^).

**Figure 7:**
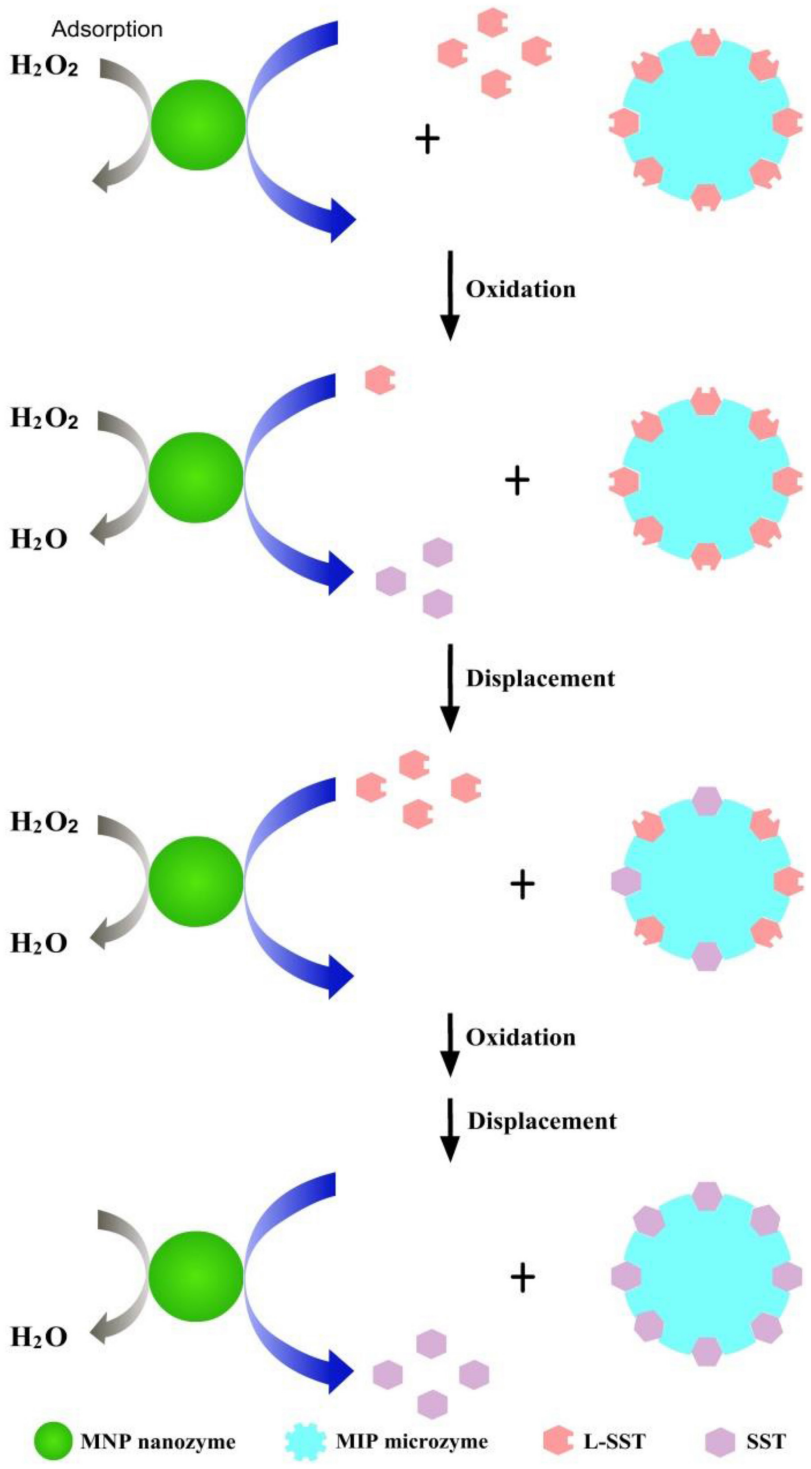
Illustration for the mechanism of the synergistic catalysis.

To reveal the mechanism of the synergistic catalysis, we first study the adsorption of H_2_O_2_ by the MNP nanozymes after an incubation of 3 min. The analytical approach for H_2_O_2_ followed a previous work (Wang et al., 2010). It is seen in Figure 6 that ~50% H_2_O_2_ was adsorbed by the MNP nanozymes after adding H_2_O_2_ to the MNP nanozymes in 3 min. Therefore, we can suggest that the oxidizing reagent is immobilized by the solid nanozymes, the cyclization of peptides is occurred when the L-SST molecules in the solution collide with the immobilized H_2_O_2_. Compared to the traditional solid phase synthesis by immobilization of the linear peptide, the reaction on the MNP nanozymes is a “reversed solid phase synthesis.” We deem the “reversed solid phase synthesis” and the traditional solid phase synthesis can inhibit the intermolecular reaction with a same way (see Figure 7).

As has been demonstrated in our previous work, the preferential binding of L-SST by the MIP microzymes play a significant role during the cyclization of L-SST: (i) The adsorption of the reactant L-SST decreases the L-SST concentration in the solution, which reduce the collision of the L-SST and the oxidizing reagent; (ii) The separation of the product SST shifts the reaction balance to the yield of SST. Therefore, we conclude the imprinted cavities of the MIP microzymes act as “hot pockets” to control the cyclization of the linear peptides (see Figure 7).

## Conclusions

In summary, we have developed a significantly efficient “polymeric microzymes and inorganic nanozymes” synergistic catalysis strategy for the formation of disulfide bonds in peptides. The integration of the two different artificial enzymes in one process facilitates the intramolecular cyclization in high product yields with excellent selectivity. Mechanism study shows the synergistic effect was occurred by using a “reversed solid phase synthesis” strategy with an enhanced shift of reaction balance to product generation. Further works based on coating of MIP microzymes onto the surface of the MNP nanozymes with peroxidase-like functions to produce binding pockets for both of the target cyclic peptide and the linear peptide are ongoing in our laboratory. We believe the synergistic catalysis by “polymeric microzymes and inorganic nanozymes” presented in the present work may open new opportunities in creation of multifunctional enzyme mimics for sensing, imaging, and drug delivery.

## Author contributions

XS designed experiments, collected samples, analyzed the data, interpreted the results and wrote the manuscript. ZC and BS helped the preparation of the manuscript.

### Conflict of interest statement

The authors declare that the research was conducted in the absence of any commercial or financial relationships that could be construed as a potential conflict of interest.
